# An unexpected case of hypercalcaemia and hilar fullness

**DOI:** 10.7196/AJTCCM.2020.v26i3.043

**Published:** 2020-10-13

**Authors:** M Laher, G A Richards

**Affiliations:** Department of Internal Medicine, Faculty of Health Sciences, University of Witwatersrand, Johannesburg, South Africa

**Keywords:** hypercalcaemia, hilar fullness, hyperparathyroidism

## Abstract

Hypercalcaemia, a condition with abnormally raised calcium levels, is commonly caused by cancer, immobility, certain supplements and other diseases such as sarcoidosis. In this case report, we present a 65-year-old female who presented with hypercalcaemia, hilar adenopathy on chest X-ray and a pathological fracture of her ankle that was unexpectedly due to hyperparathyroidism.

## Background

A 65-year-old female presented with a fracture of her left ankle following a motor vehicle collision. She required anticoagulation as she developed a deep-vein thrombosis as a result of her immobility. She had been complaining of generalised bone pains over the past few months prior to the event, which she had attributed to old age. As radiological evidence of significant osteoporosis was found, a conservative management approach was adopted for the fracture.

She was clinically well and showed no evidence of bleeding or skin rashes on examination. There was swelling around her ankle with decreased range of motion. There was no evidence of neurological or vascular compromise. The respiratory system examination was normal.

**Fig. 1:**
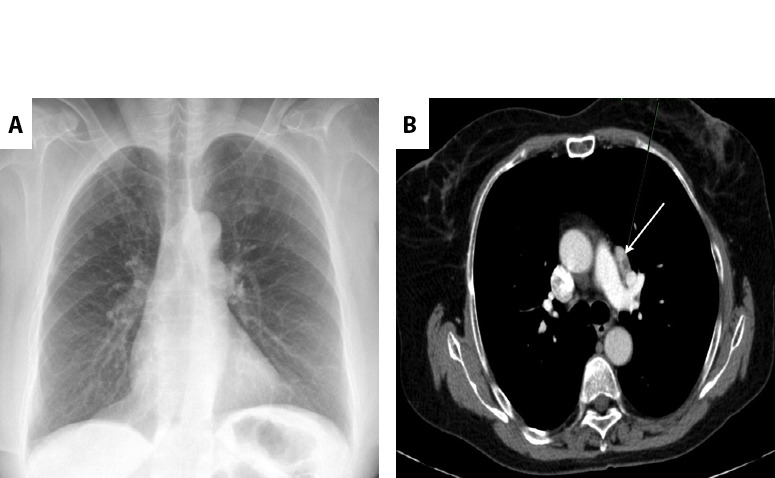
Chest X-ray (A) and computed tomography scan (B) revealing hilar fullness.

Her chest X-ray revealed hilar fullness with the left being more prominent than the right (Fig. 1).

Laboratory investigations (normal ranges) showed that calcium was 2.71 (2.20 - 2.55) mmol/L, phosphate was 0.68 (0.78 - 1.42) mmol/L, parathyroid hormone (PTH) was 36.3 (1.60 - 6.0) pmol/L and alkaline phosphatase was 127 (42 - 98) U/L. She had normal renal function (glomerular filtration rate >60 mL/min/1.73 m^2^ ) and serum angiotensin converting enzyme level of 23 U/L. Her total vitamin D level was at the lower limit of normal at 73.08 nmol/L (sufficient level >72.5 nmol/L).

A parathyroid sestamibi scan revealed an area of hyperplasia within the mediastinum. A computed tomography scan confirmed the presence of a lesion situated at the left hilum. No enlarged parathyroid glands were observed in the neck. A dual energy X-ray absorptiometry scan confirmed the presence of osteoporosis with a t-score of –4.3 in the spine and –4.8 in the hip

She received a dose of zoledronic acid for the osteoporosis and saline diuresis pre-operatively to decrease the serum levels of calcium. She underwent video-assisted thoracoscopic surgery (VATS) to remove the mediastinal mass. Following this, the serum calcium levels decreased to 2.19 mmol/L and the PTH decreased to 15.6 pmol/L. The histology confirmed the presence of ectopic parathyroid tissue that had atypical cells with clear and eosinophilic cytoplasm and stippled chromatin with the round and uniform nuclei. The atypical endocrine cells stained positive for chromogranin, synaptophysin and parathyroid hormone.

Unfortunately, she developed a post-operative complication – on re-initiation of therapeutic anticoagulation, she bled from the vessel supplying the mass. She developed a haemothorax which required drainage as well as a blood transfusion but made a full recovery thereafter.

## Discussion

The differential diagnosis of a hilar mass requires exclusion of possible causes such as infectious pathogens, cancer (carcinomas or lymphomas) and sarcoidosis. The coexistence of a hilar mass with hypercalcaemia may suggest that the latter two are the more likely causes.^[1]^

Hyperparathyroidism is an underdiagnosed condition in the developing world. In the South African setting, the median age is 60 years with a female predominance of 78.6%. Most of the patients are symptomatic and surgery remains the mainstay of therapy.^[2]^

Ectopic lesions occur in up to 20% of all cases of parathyroid adenoma^[3]^ and always pose a treatment and diagnostic challenge. A combination of radiological investigations have been utilised to increase the sensitivity and specificity of the diagnosis and facilitate the localisation of these lesions prior to surgical removal. Accurate localisation has improved the success of minimally invasive surgery (VATS) and has reduced the need for median sternotomy to reach these hard-to-locate lesions.^[4]^ Although a diagnosis might be suspected, histological confirmation is required to make a correct diagnosis

The majority of ectopic glands are found in the mediastinum, which is related to its embryonic development. When ectopic tissue descends into the mediastinum, it does so with the thymus during the fifth week of development. It is usually located below the level of the clavicle, with the deeper ones posing more of a challenge. The inferior glands are located in the anterior mediastinum, while the rarer superior glands are found in the posterosuperior mediastinum.^[5]^

In this case, the cause for hypercalcaemia was unexpected and not one usually included in the differential possibilities of a respiratory physician.
